# Preclinical trial of a MAP4K4 inhibitor to reduce infarct size in the pig: does cardioprotection in human stem cell-derived myocytes predict success in large mammals?

**DOI:** 10.1007/s00395-021-00875-7

**Published:** 2021-05-20

**Authors:** Maaike te Lintel Hekkert, Gary Newton, Kathryn Chapman, Rehan Aqil, Robert Downham, Robert Yan, Daphne Merkus, Gavin Whitlock, Charlotte A. L. Lane, Darren Cawkill, Trevor Perrior, Dirk J. Duncker, Michael D. Schneider

**Affiliations:** 1 Department of Cardiology (Thoraxcenter), Erasmus University Medical Center, University Medical Center Rotterdam, PO Box 2040, 3000 CA Rotterdam, The Netherlands; 2 Domainex Ltd, Saffron Walden, UK; 3 Sandexis Medicinal Chemistry, Sandwich, UK; 4 Apollo Therapeutics, Stevenage, UK; 5 National Heart and Lung Institute, Imperial College London, Du Cane Road, London, W12 0NN UK

**Keywords:** Cardioprotection, Drug development, Human pluripotent stem cell-derived cardiomyocytes, Infarct size

## Abstract

Reducing infarct size (IS) by interfering with mechanisms for cardiomyocyte death remains an elusive goal. DMX-5804, a selective inhibitor of the stress-activated kinase MAP4K4, suppresses cell death in mouse myocardial infarction (MI), human pluripotent stem cell-derived cardiomyocytes (hPSC-CMs), and 3D human engineered heart tissue, whose fidelity to human biology is hoped to strengthen the route to clinical success. Here, DMX-10001, a soluble, rapidly cleaved pro-drug of DMX-5804, was developed for i.v. testing in large-mammal MI. Following pharmacodynamic studies, a randomized, blinded efficacy study was performed in swine subjected to LAD balloon occlusion (60 min) and reperfusion (24 h). Thirty-six animals were enrolled; 12 were excluded by pre-defined criteria, death before infusion, or technical issues. DMX-10001 was begun 20 min before reperfusion (30 min, 60 mg/kg/h; 23.5 h, 17 mg/kg/h). At all times tested, beginning 30 min after the start of infusion, DMX-5804 concentrations exceeded > fivefold the levels that rescued hPSC-CMs and reduced IS in mice after oral dosing with DMX-5804 itself. No significant reduction occurred in IS or no-reflow corrected for the area at ischemic risk, even though DMX-10001 reduced IS, expressed in grams or % of LV mass, by 27%. In summary, a rapidly cleaved pro-drug of DMX-5804 failed to reduce IS in large-mammal MI, despite exceeding the concentrations for proven success in both mice and hPSC-CMs.

**Supplementary Information:**

The online version contains supplementary material available at 10.1007/s00395-021-00875-7.

## Introduction

The pandemic burden of ischemic heart disease [12] and acute myocardial infarction (MI) has driven diverse efforts to rescue cardiac muscle cell number, as the extent of muscle loss drives later progression to heart failure [27]. Urgent reperfusion to restore coronary flow is the standard of care for MI but paradoxically confers tissue damage itself, by reintroducing oxygen to ischemic regions and inducing mitochondrial reactive oxygen species. To address the unmet need for effective cardioprotection as an adjunct to reperfusion, multiple therapies have been proposed, yet successful translation to humans has been elusive [17]. Among factors contributing to this gap stands the absence, historically, of efficacy testing in any human model system. Thus, the emerging use of human cardiomyocytes from pluripotent stem cells (hPSC-CMs) is potentially transformative as a robust, scalable platform to enhance cardiac drug discovery [13, 14]. Possible short-comings of this experimental tool are acknowledged, however, and its predictive power better proven, presently, for safety pharmacology than for novel therapeutics [10, 13, 14].

Mitogen-activated protein kinase kinase kinase kinase-4 (MAP4K4) is an upstream member of the MAP kinase superfamily that is activated in failing human hearts and rodent models of cardiac cell death including ischemia–reperfusion injury in vivo and oxidative stress in vitro [11]. Small-molecule inhibitors of MAP4K4 were devised through chemical library screening, pharmacophore similarity analysis, and structure-driven drug design [11]. The compound DMX-5804 suppressed death from oxidative stress in hPSC-CMs, blocked cell death in engineered human heart tissue, and reduced infarct size (IS) in mice by > 50%, even given an hour after reperfusion [11]. From this proof-of-principal support—not just in small-mammal studies but also hPSC-CMs in 2- and 3D culture—MAP4K4 is a prudent target for progression toward the objective of limiting human cardiac cell death. DMX-5804 itself already possessed many favorable properties, including potency, bioavailability after gavage, and stringent selectivity, interfering potently just with MINK1/MAP4K6 and TNIK/MAP4K7, among 376 kinases [11]. However, its aqueous solubility was inadequate for i.v. delivery, as envisioned for urgent treatment of MI in humans.

Large mammals are indispensable in cardiac drug discovery, not merely for the proof of safety that regulators demand but also the value of proving effectiveness in a model closer to humans than those already mentioned. The pig is especially noteworthy, showing fidelity in heart size, heart rate, coronary anatomy, collateral vessels, timecourse of damage, post-ischemic immune response, and compatibility with clinically relevant interventions and protocols [18].

Here, we report a novel, highly soluble analog of DMX-5804, designated DMX-10001, that is suitable for i.v. administration and evaluation in an established porcine model of MI. The concentrations predicted to be therapeutic were achieved. However—notwithstanding prior success in mice, hPSC-CMs, and 3D engineered human heart tissue—no significant reduction was seen in IS or no-reflow (NR) corrected for the area at ischemic risk (AAR).

## Methods

### Medicinal chemistry

**Fig. 1:**
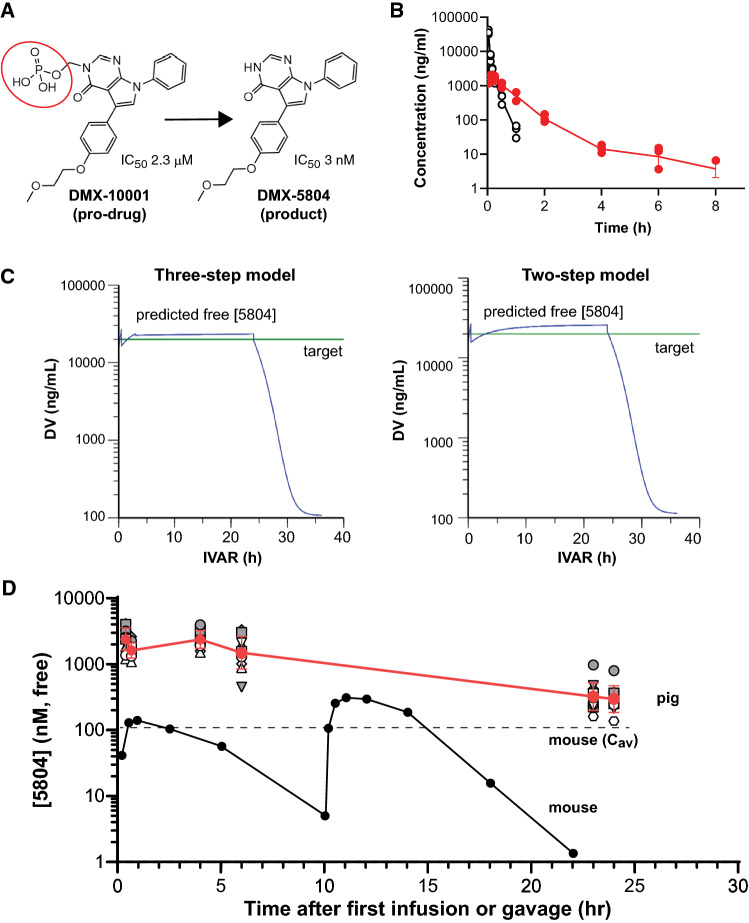
Predicted and verified levels of the active agent DMX-5804, following infusion of DMX-10001. **a** Chemical structures, highlighting the phosphate moiety of the pro-drug. **b** Rat PK after i.v. injection of 5 mg/kg DMX-10001, showing rapid disappearance of the pro-drug and generation of DMX-5804 even at the earliest time-points. Data are the geometric mean ± SD; *n* = 3. **c** Predicted levels in pigs from three- and two-step infusion protocols (Left: 60 mg/kg/h, 0.5 h + 20 mg/kg/h, 2.5 h + 16 mg/kg/h, 21 h. Right: 60 mg/kg/h, 0.5 h + 17 mg/kg/h, 23.5 h.) DV, dependent variable (ng/mL); IVAR, independent variable (*h*); green line, target concentration. **d** Empirically determined levels of DMX-5804 in the efficacy study, using two-step infusion as in (**c**). The geometric mean ± SD (red) and individual levels in all 12 treated swine (white or gray) are shown, by comparison with the serial levels (black) and time-averaged concentration in mice (*C*_av_) that reduced infarct size by 50%. Mouse PK results are from ref. [11]. Results are shown relative to the time of first dosing, which differed between the species (pig, 20 min before reperfusion; mouse, 60 min after reperfusion)

DMX-10001, a rapidly hydrolyzed phosphate pro-drug of DMX-5804, was produced by Domainex Ltd, by a modification of the latter’s synthetic route [11], attaching a hydrolysable phosphate group via a linker to the core of DMX-5804 (Fig. 1a). This removes a key hydrogen bonding group and prevents DMX-10001 from binding to the hinge region of the MAP4K4 kinase domain. In cell-free biochemical assays, DMX-5804 has an IC_50_ of 3 nM against MAP4K4; whereas, DMX-10001 has an IC_50_ of 2.3 µM, more than a 750-fold difference. Hence, DMX-10001 is essentially inactive against MAP4K4 and its conversion to DMX-5804 is required for activity. Furthermore, this modest value is likely driven by minute levels of DMX-5804 either present in the sample or generated during by hydrolysis during the assay. In short, the pro-drug DMX-10001 will not significantly contribute to inhibition of MAP4K4, itself.

Compound concentrations after dosing were measured as follows by liquid chromatography–mass spectrometry (Pharmidex, London, UK). To 50 µL aliquots of plasma was added 200 µL of internal standard solution (methanol containing 500 ng/mL tolbutamide). Samples were then mixed (150 rpm, 20 min) and centrifuged (3000 rpm, 15 min). 75 µL of the resulting supernatant was added to 75 µL 10 mM ammonium acetate + 0.5% ammonia) and mixed (450 rpm, 15 min). Calibration standards covering the expected concentration range were prepared by spiking analyte into control matrix, with extraction and analysis in the same way as the plasma samples.

Analysis was done by liquid chromatography–mass spectrometry using a Shimadzu Nexera X2 ultra-high-pressure liquid chromatography system coupled to a Shimadzu LCMS 8060 triple quadruple mass spectrometer (Shimadzu, Milton Keynes, UK) with Waters Acquity BEH C18 (50 × 2.1)mm, 1.7 µm column (Waters, Elstree, UK) and mobile phase components 10 mM ammonium acetate + 0.5% ammonia (A) and methanol + 0.5% ammmonia (B). Gradient was 0–0.5 min 30% B; 0.5 to 2.5 min increase to 80% B; 2.5–2.6 min increase to 98% B; 2.6–3 min 98% B; 3–3.1 min decrease to 30% B; 3.1–3.5 min 30% B. Flow rate was 0.4 mL/min. Injection volume was typically 5 µL. Detection was by multiple reaction monitoring, DMX-5804 361.9 > 304.1 (positive ion), DMX-10001 470.1 > 258.0 (negative ion), and tolbutamide 271.0 > 91.0 (positive ion). Retention times were DMX-5804 2.9 min, DMX-10001 2.1 min, and tolbutamide 1.8 min. Data processing was done by Shimadzu LabSolutions Software (Shimadzu, Milton Keynes, UK).

Results were corrected for plasma protein binding by rapid equilibrium dialysis to calculate free drug levels, and were subjected to iterative computer modeling (XenoGesis, Alderley Park, UK) using Phoenix64 WinNonlin (Certara, Princeton, NJ), to devise the infusion protocol.

### Animals

This study was submitted for registry at https://preclinicaltrials.eu on November 7, 2019 and published January 15, 2021 (PCTE0000143). Pig studies were performed in Yorkshire × Landrace swine of either sex (Rovar Woensdrecht b.v., Woensdrecht, The Netherlands), in accordance with the “Guiding Principles in the Care and Use of Laboratory Animals” as approved by the Council of the American Physiological Society, and with approval of the Animal Care Committee of the Netherlands (AVD1010020181665).

Early PK studies were performed in male Han Wistar rats by Saretius (Reading, UK), in accordance with the UK Animals (Scientific Procedures) Act 1986 and the terms of Licence 70/8420 and with approval of the University of Reading Local Ethical Review Process.

### Pilot physiological study

Pilot physiological studies were performed in 2 female swine (45 and 85 kg), to confirm the lack of adverse effects from DMX-10001 in healthy myocardium. Animals were chronically instrumented as detailed below [6]. Following a recovery week, animals received the pro-drug DMX-10001, while hemodynamics were recorded. Based on prior pharmacokinetic studies and modeling (Fig. 1b), two different dosing regimens were investigated, a three-step infusion (41 mg/kg/h, 0.5 h + 15.3 mg/kg/h, 2.5 h + 9.8 mg/kg/h, 5 h) versus a logistically simpler two-step approach (60 mg/kg/h, 0.5 h + 17 mg/kg/h, 23.5 h). During infusion of the pro-drug, blood was sampled to measure production of the active form, DMX-5804, by liquid chromatography–mass spectometry (Pharmidex, London, UK) as described [11].

### Efficacy study

Next, an efficacy study was performed, in adherence to published guidelines for rigor and reproducibility [2], to evaluate the postulated benefit of DMX-10001 in a large-mammal model of MI. Briefly, 36 closed-chest swine (41 ± 2 kg) were subjected to regional ischemia by occluding the left anterior descending coronary artery (LAD) distal to the first diagonal branch for 60 min, followed by 24 h of reperfusion [7, 22, 24]. Treatment with DMX-10001 (60 mg/kg/h for 0.5 h, followed by 17 mg/kg/h for 23.5 h) versus the vehicle was initiated 20 min prior to reperfusion and continued for 1 day. After 2 h of reperfusion, animals were allowed to recover from anesthesia. At 24 h of reperfusion, the infarct area (IA), area of no-reflow (NR), and area at ischemic risk (AAR) were determined [30, 31, 33]. Transthoracic short-axis B-mode echocardiograms and measurements of LV pressure and its first derivative (LVd*P*/d*t*) were obtained at baseline and at 24 h of reperfusion. LV pressure–volume measurements were obtained at 24 h of reperfusion. Heart-type fatty acid binding protein (hFABP) release was measured, as a biomarker that correlates with IS and NR in swine at least as well as cardiac troponin I [30].

### Blinding and exclusions

Investigators involved in the experimental protocol and analysis of the data were blinded to the treatment status of each pig. Animals were randomly assigned to the treatment groups in two blocks, using a random number series generated online. To control further for potential investigator bias, inclusion and exclusion criteria were formulated prior to the onset of the study. Only healthy pigs weighing 38–48 kg, with a body temperature between 37.0 and 40.0 °C at the start of surgical preparation, were included. Exclusion criteria were: (i) Encountering ventricular fibrillation (VF) during the experimental protocol that could not be converted to sinus rhythm within 2 min. (ii) Encountering VF > 4 times before starting the drug or vehicle infusion. (iii) Developing progressive pump failure (mean arterial pressure (MAP) < 30 mmHg) during first 30 min of reperfusion. (iv) Incomplete coronary artery occlusion (CAO) at the end of occlusion, verified by contrast dye injection. (v) Aberrant anatomy, producing technical difficulties that prevented successful catheterization. (vi) AAR < 15% of the LV. During the study, one animal exhibited VF before the start of the ischemia–reperfusion protocol and, hence, prior to any infusion of placebo or drug. This was not foreseen in the initial design of the exclusion criteria and was not then an exclusion criterion. However, in view of the risk of preconditioning the heart during this episode of VF, we chose to stop the procedure and add the further criterion “Encountering VF prior to the onset of the ischemia–reperfusion protocol.” Following any exclusions as above, modifications were made where necessary to ensure that the two treatment groups remained appropriately balanced for sex and weight. See also Data and statistical analysis, below.

### Surgical preparation

After overnight fast, swine were sedated with an i.m. injection of tiletamine/zolazepam (6 mg/kg, Zoletil50, VIRBAC Nederland BV, Barneveld, The Netherlands), xylazine (2.25 mg/kg, Sedazine10%, AST Farma, Oudewater, The Netherlands) and atropine sulfate (0.03 mg/kg, Teva Nederland BV, Haarlem, The Netherlands) [6], anesthetized with thiopental sodium (4 mg/kg, ROTEXMEDICA GmbH, Trittau, Germany), intubated, and mechanically ventilated with a mixture of O_2_ and air (FiO_2_ 23–35%, EtCO_2_ 4–6 kPa, PEEP 4 cm H_2_O). Anesthesia was maintained with i.v. administration of sufentanil (5 µg/kg/h, Sufenta Forte, Hameln Pharma plus GmbH, Hameln, Germany) and midazolam (0.5 mg/kg/h, Actavis, Baarn, The Netherlands). Saline was infused at 200 ml/h i.v. to maintain fluid status of the animals and physiological body core temperature was maintained between 37.1 and 38.1 °C during CAO with ice or heating pads [8]. Antibiotic prophylaxis was administered, consisting of a mixture of procainebenzyl penicillin and dihydrostreptomycine sulfate (20,000 IU/kg and 20 mg/kg, i.m., Instruvet Nederland bv, Boxmeer, The Netherlands).

Under sterile conditions, the left carotid artery was cannulated and 10,000 IU of heparin (LEO Pharma, Amsterdam, The Netherlands) were administered to establish anticoagulation, followed by 5000 IU every additional h. During surgical preparation and the experimental protocol, aortic blood pressure and ECG were continuously recorded. At 15 min of reperfusion, i.v. anesthetics were reduced (midazolam was stopped, sufentanil was halved) and a gas anesthetic was added (sevoflurane 1–2% v/v, AbbVie bv, Hoofddorp, The Netherlands); at 1 h of reperfusion sufentanil was stopped. This protocol was chosen to obviate the confounding cardioprotective effects of inhalation anesthetics if given prior to ischemia or in the first few minutes of reperfusion. The two fluid-filled catheters were kept in place, one in the aorta for blood sampling and one in the vena cava for DMX-10001 or vehicle infusion. These were tunneled subcutaneously to exit through the back of the animal and protected with a vest. After the wound was closed, the animals were allowed to recover. No analgesics were given, due to the minor surgical intervention.

The next day, swine were sedated, anesthetized and ventilated as above, except that the amount of xylazine (1.12 mg/kg) and atropine sulfate (0.015 mg/kg) was halved. Anesthesia was maintained with i.v. sufentanil (5 µg/kg/h) and midazolam (0.5 mg/kg/h). Saline was infused at 200 ml/h i.v. to maintain the animals’ fluid status.

### Hemodynamics and LV function

A transthoracic short-axis B-mode echocardiogram (ZS3, Zonare Medical Systems, Mountain View, CA) was performed to determine LV contractile performance. LV pressure and its first derivative (LVd*P*/d*t*) were determined using a micro-manometer-tipped catheter (SPR-350S, Millar Inc., Houston, TX), before infarct induction and again before killing. Terminal LV pressure–volume measurements were also performed, using a 7F conductance catheter (CD Leycom, Hengelo, The Netherlands) advanced into the LV [32]. After calibration of the system, pressure–volume loops were recorded during a breath hold.

### Ischemia–reperfusion

The LAD was catheterized with a standard clinical guiding catheter (JL3.5, 6F, Medtronic, Minneapolis, MN), and quantitative coronary angiography (CAAS, PIE Medical, Maastricht, The Netherlands) was performed following 2 mg isosorbidedinitrate (Cedocard, Nycomed, Hoofddorp, The Netherlands) and using iodixanol as contrast agent (Visipaque™, GE Healthcare BV, Eindhoven, The Netherlands) [30]. Then, an over-the-wire coronary angioplasty balloon (Sprinter OTW, 2–3 mm × 6 mm, Medtronic, Minneapolis, MN) on a standard guide wire (0.014″, 180 cm, ASAHI SION blue, ASAHI Intecc co Ltd, Aichi, Japan) was carefully positioned under fluoroscopic guidance distal to the first diagonal branch. Following balloon inflation, total coronary occlusion was confirmed by angiography (at baseline and at 58 min of occlusion) and by ECG changes. Animals that developed VF during the protocol were defibrillated within 25 ± 10 s. After 1 h of occlusion, the coronary balloon was deflated to establish reperfusion, which was angiographically confirmed to ensure an adequate coronary blood flow to the entire LAD perfusion territory. Heart rate, aortic blood pressure, and ECG were monitored until 2 h of reperfusion.

### Drug administration

DMX-10001 was dissolved in 10 mM PBS, pH 7.6, and administered via a fluid-filled catheter in the inferior vena cava versus vehicle (10 mM PBS, pH 7.4) in the control animals. Twenty min before the onset of reperfusion, an initial rapid administration was started (30 min infusion, 60 mg/kg/h, Alaris CC, Cardinal Health, Role, Switzerland), followed after 10 min of reperfusion by a lower sustained dose (23.5 h infusion, 17 mg/kg/h, Myfuser, CANOX medical Device Srl, Capurso, Italy). The dosing regimen was determined by iterative computer modeling (XenoGesis Ltd, Alderley Park, UK) (Fig. 1b), based on prior dose-finding studies in pigs (data not shown).

For blinding, all solutions of DMX-10001 and vehicle were prepared and administered by someone not involved in the experimental procedures or analysis of results.

### Determination of AAR, infarct size and area of no-reflow

After completing the post-infarct LV functional measurements on day two, the LAD was again catheterized, as before. The over-the-wire coronary angioplasty balloon and guide wire were positioned under fluoroscopic guidance at the same position, using anatomic landmarks (distance from sidebranches) visualized by coronary angiography. Through the lumen of the balloon, 5 ml of 4% (*w*/*v*) thioflavin-S solution (Sigma, Zwijndrecht, The Netherlands) were injected, allowing the area of no-reflow within the AAR to be measured later histologically. The balloon was re-inflated to re-occlude the LAD and a sternotomy was performed. Next, a bolus of 40 ml of a 15% (*w*/*v*) Evans Blue solution was injected into the left atrium for negative staining of the AAR. To facilitate excision, the heart was arrested via induction of VF by gently touching the LV epicardium with a 9 V battery. The LV was isolated and sectioned into 5 transverse slices. The AAR and area of no-reflow (NR), visualized using UV light, were quantified in all sections [15, 30, 31, 33]. Cardiac slices were subsequently incubated in a 1% solution (*w*/*v*) 2,3,5-triphenyltetrazolium chloride (Sigma, Zwijndrecht, The Netherlands) at 37 °C for 15 min to discriminate between metabolically active and dead myocardium, and macroscopically assess infarct size (IS). Total LV size, AAR, IS and NR in g were calculated, after correcting for slice weight [25].

### Analysis of blood samples

**Fig. 2:**
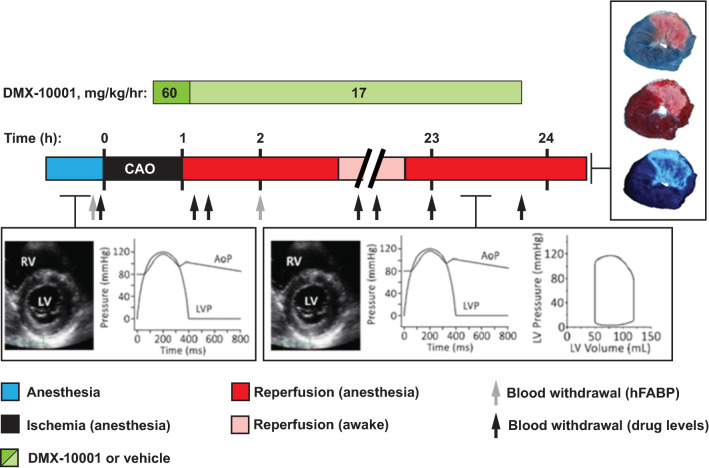
Experimental protocol. Swine underwent 60 min of LAD occlusion, followed by 24 h of reperfusion, and were treated with vehicle versus DMX-10001 using a two-step infusion protocol. Hemodynamics and LV function were measured as shown beneath the timeline. Animals were then euthanized and analyzed for IS, NR, and AAR

At several time-points (see Fig. 2), blood samples were taken in lithium-heparin-coated tubes (BD, Plymouth, UK) to analyze plasma levels of DMX-10001 (pro-drug) and DMX-5804 (active compound) and in EDTA-coated tubes (BD, Plymouth, UK) to measure heart-type fatty acid binding protein (hFABP). All blood samples were kept on ice immediately after withdrawal and were centrifuged for 10 min at 3000 rpm and 4 °C within 6 h. Plasma was aliquoted and cryopreserved at − 80 °C until analysis. Levels of DMX-10001 and DMX-5804 were determined by liquid chromatography–mass spectrometry (Pharmidex, London, UK). Porcine hFABP was analyzed as described [30]: plasma samples were thawed on ice, diluted 10 × using the standard diluent provided with the kit, and analyzed by enzyme-linked immunosorbent assays according to the manufacturer’s instructions (Life Diagnostics, West Chester, PA). Absorbance was measured at 450 nm with a microplate photometer (Multiskan EX, Thermo Scientific, Etten-Leur, The Netherlands).

### Data and statistical analysis

Values are expressed as the mean SD for all results, excepting the geometric mean ± SD for pharmacokinetics. All data were normally distributed. Inter-group differences, except as below, were analyzed using the unpaired two-tailed *t* test (Prism 9.0, GraphPad Software, San Diego, CA or IBM SPSS Statistics 25, Armonk, NY) and by analysis of covariance (ANCOVA), with AR/LV, AR (g) or IA/AR as a covariate (SAS, version 9.4). The full statistical analysis of these data was performed by an independent external consultant, Kat Gore (KDL Gore Solutions Ltd). As part of the a priori work plan, if large statistical outliers were identified in the analysis diagnostic plots, those outliers were excluded from a repeat analysis, and any potential changes in conclusions were summarized.

Sample size calculation was performed for a minimal detectable difference in IS/AAR of 20%, expected SD of 16.5, alpha of 0.05 and power of 0.8, yielding a required n of 11. Taking into account a dropout rate of 30% due to peri- and post-infarct mortality (VF or pump failure during ischemia and reperfusion) plus a 10% dropout due to technical issues, a total number of 18 animals per group were required. As noted above, in our actual study, the combined dropout rate was just 33% (less than the predicted 40%), for an *n* of 12 swine per group.

Hemodynamic and LV global function were recorded and digitized online using a three-channel data acquisition program (ATCODAS, Dataq Instruments Inc., Akron OH) and stored on a computer for off-line analysis with a program written in MATLAB (Mathworks Inc., Natick, MA). A minimum of 10 s was selected for analysis of the digitized hemodynamic signals. Pressure–volume-related data were recorded and stored on a computer for off-line analysis (CD Leycom, Hengelo, The Netherlands). Statistical significance (*P* < 0.05, two tailed) for changes was determined by two-way ANOVA (time point in protocol x treatment) for repeated measures, followed by post hoc testing with the Bonferonni multiple comparison test.

## Results

### A highly soluble pro-drug of DMX-5804, suitable for i.v. delivery

The chemical structures of DMX-5804 and DMX-10001 are compared in Fig. 1a, highlighting the phosphate moiety of the pro-drug. The aqueous solubility of DMX-10001 was > 400-fold greater than that of DMX-5804, allowing it to be infused i.v. without prohibitive fluid volumes. Cleavage to DMX-5804 was detectable in rats within 5 min and largely complete within 60 min (Fig. 1b). Data from i.v. infusion at a range of dose levels in the pig were simultaneously fit to a three-compartment pharmacokinetic model. Plasma protein binding in pig was used to determine free drug levels (not shown). Various dosing regimens were simulated to avoid an initial spike in DMX-5804 whilst rapidly achieving the efficacious drug levels seen in mice after oral gavage (Fig. 1c).

No adverse effect on hemodynamics or LV function was seen in pilot studies of two healthy animals (Online Tables 1 and 2).

### Enrollment and exclusions

For efficacy testing, shown schematically in Fig. 2, the enrollment and exclusions are summarized in Online Table 3. In all, 36 animals were enrolled, of which 24 were included in the final analysis. Animal weight and male/female distribution did not differ between the treatment groups. The total percentage of animals that could not be included amounted to 33%, which comprised exclusion either due to premature death or due to technical issues. Six out of 36 animals died following infarct induction, due to non-convertible VF (2) or pump failure (4: 1 died during recovery from anesthesia, 1 died overnight, and 2 died following sedation on day 2). A further 6 animals were excluded from the outcome analysis arising from technical complications: 1 experienced VF during surgical instrumentation, i.e., before onset of the experimental protocol, 2 were excluded due to technical failure with the balloon catheter during the experimental protocol, 2 were excluded due to complications during tissue staining for the assessment of the area at risk), and 1 had an area at risk that was smaller than 15% of the left ventricle. Of the included animals, 14 encountered VF during the 60 min of CAO, all prior to treatment (vehicle, 7/12, 58%; DMX-10001, 7/11, 64%; *P* = 0.77; Online Table 4). Sinus rhythm was restored within 62 ± 17 s in the vehicle group and 69 ± 34 s in the DMX-10001 group (*P* = 0.68); consequently, all animals were eligible to continue the ischemia–reperfusion protocol. Likewise, the number/animal of VF episodes (*P* = 0.34) and DC shocks (*P* = 0.74) did not differ between groups (Online Table 4). VF did not occur during the subsequent drug infusion.

### Pharmacokinetics

Levels of DMX-5804 after infusion with DMX-10001 corresponded well with those anticipated from computer modeling (Fig. 1c, d). The target level sought, based on successful reduction of IS in mice [11], was achieved in all animals, with high exposure even at the earliest timepoint and throughout the critical first 6 h. Throughout, the free concentrations of DMX-5804 from pro-drug infusion in pigs (free AUC_0-24_, 27,900 h.nM; free *C*_av_ 1,160 nM) were higher than those in mice after twice daily oral dosing of DMX-5804 itself (free AUC_0–22_, 2,200 h.nM; free *C*_av_, 1,160 nM) [11]. Thus, exposure to DMX-5804 was achieved by i.v. delivery of the pro-drug, surpassing the concentrations needed to reduce cell death in mouse MI and hPSC-CMs (e.g., EC_50_ 500 nM in vCor.4U cells treated with 400 μM H_2_O_2_ [11]).

### Hemodynamics and LV contractile performance

Heart rate and arterial pressures in the efficacy study are summarized in Online Table 5. CAO provoked similar decreases, which recovered partly during reperfusion in both treatment groups. LV function was determined using transthoracic short-axis B-mode echocardiography, a micro-manometer-tipped catheter, and conductance catheter measurements (Online Tables 6–8). As expected, ischemia–reperfusion injury impaired LV dimensions, d*P*/d*t*_min_, tau and end-diastolic pressure, 2D ejection fraction, 2D wall thickening in the infarct region, end-systolic pressure, d*P*/d*t*_max_ and d*P*/d*t*_P40_. These infarct-related functional changes were comparable to those in prior pig studies [21]. DMX-10001 neither worsened nor enhanced these physiological end-points.

### IS and no-reflow

**Fig. 3:**
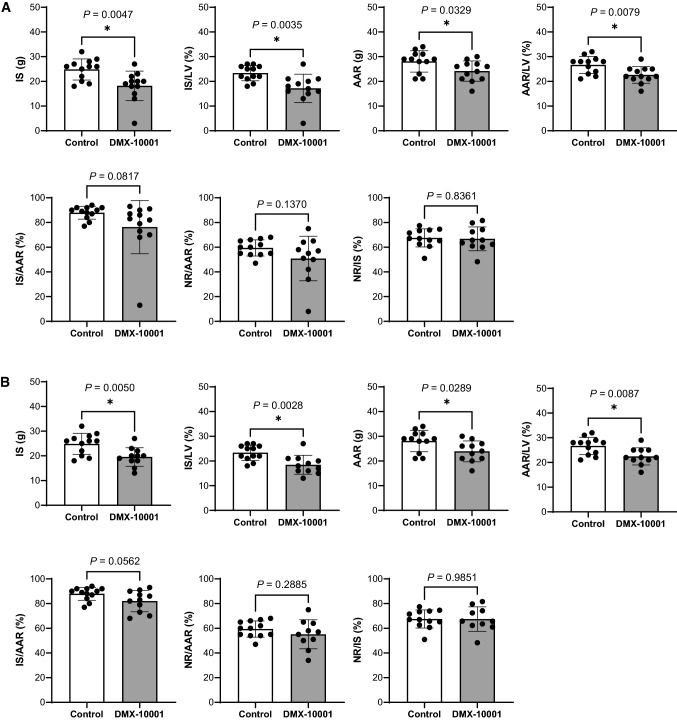
Effects of DMX-10001 infusion on IS and NR. **a** Data are shown as single data points plus the mean ± SD. Vehicle, *n* = 12; DMX-10001, *n* = 12; *P < 0.05; unpaired two-tailed *t* test. Absolute IS and the IS/LV ratio were reduced by DMX-10001, attributable to an underlying difference in AAR. **b** Data as in (**a**), omitting an outlier that was well beyond the standard deviation for DMX-10001-treated animals. The conclusions were identical in both analyses

As expected, no significant difference between groups was seen in LV mass (vehicle, 105.5 ± 9.0 g; DMX-10001, 106.9 ± 9.7 g). For the full cohort of 24 animals (vehicle, 12; DMX-10001, 12), the extent of infarction in grams or % of LV mass was reduced 27% by DMX-10001 (24.8 ± 4.3 vs 18.2 ± 5.8 g, *P* = 0.0048; 23.4 ± 3.1 vs 17.2 ± 5.7%, *P* = 0.0035; Fig. 3a). These encouraging results were largely attributable, however, to an underlying difference between groups in the AAR and AAR/LV (e.g., vehicle, 26.6 ± 3.6%; DMX-10001, 22.6 ± 3.4%; *P* = 0.0079). In vehicle-treated swine, correcting for the region at risk, CAO for 60 min provoked an IS equalling 88.0 ± 5.1% of AAR (95% CI 83.7–92.3%), and NR was 59.3 ± 6.4% of AAR. With normalization, DMX-10001 resulted in just a 13% reduction in mean IS/AAR, to 76.3 ± 21.6% (95% CI 66.9–85.7%), which did not achieve statistical significance (*P* = 0.0817; Fig. 3). There was no significant effect on NR, NR/AAR or NR/IS. Weight was explored as a potential covariate but did not improve the statistical analysis.

One animal that received DMX-10001 was identified as a statistical outlier, with a much smaller IS/AAR than all other animals (Fig. 3b). The analysis was repeated excluding that animal, as planned a priori by the independent statistician, with no difference in the conclusions (Fig. 3c): only a very small difference in IS/AAR was seen (82.1 ± 8.6%; 7% less than the vehicle control), at the threshold of significance (*P* = 0.0562). By either analysis, the study was adequately powered to resolve a 20% reduction of IS/AAR, and failed to meet this pre-specified end-point.

**Fig. 4:**
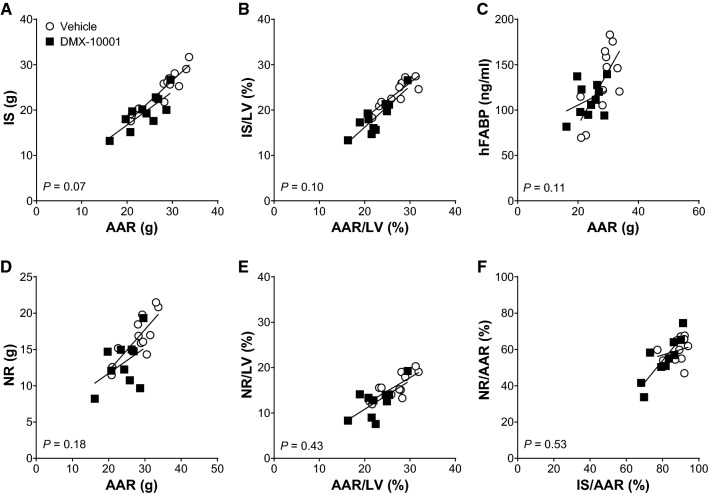
Analysis of covariance**.** The end-points were correlated with AAR, AAR/LV or IS/AAR, as shown, assuming a linear relationship. Each data point represents one animal; vehicle (open circles), *n* = 12; DMX-10001 (closed squares), *n* = 11. No significant difference in slope was seen between groups in any of these relationships

A statistically robust alternative to just dividing by AAR is analysis of covariance (ANCOVA), which provides greater protection against baseline imbalance [26]. For each treatment, the linear relationships were plotted between IS and NR as a function of AAR, as well as NR as a function of IS, and the slopes were compared. No significant differences were conferred by DMX-10001 (Fig. 4).

### hFABP release

No difference between groups was seen in baseline plasma levels of hFABP (vehicle, 9 ± 5 ng/ml; DMX-10001, 8 ± 2 ng/ml, *P* = 0.55). As expected, hFABP release was increased at 60 min of reperfusion (vehicle, to 140 ± 39 ng/ml). In agreement with the histological findings, levels with DMX-10001 were not significantly less (120 ± 19 ng/ml; *P* = 0.13).

## Discussion

In this translational study, a novel i.v. MAP4K4 inhibitor, DMX-10001, was evaluated in a large-mammal model of MI. This highly soluble, rapidly cleaved pro-drug achieved target levels of the active product, DMX-5804, in all treated animals that significantly exceeded the exposure achieved in the prior, efficacious mouse study [11]. However, the resulting benefits in pigs were small, relative to the target sought (20% reduction of IS/AAR), and lacked statistical significance. Hence, the available evidence fails to translate the efficacy of MAP4K4 inhibition from rodents into a meaningful effect in the more clinically relevant large mammal. While not surprising inherently, this shortfall is more striking given prior success not merely in mice but also in hPSC-CMs and even 3D engineered human heart tissue [11].

The present investigation sought to take these prior findings forward into a porcine, closed-chest model of MI, given wide acceptance of this model’s similarities to humans [18], and the common failure of rodent models to be predictive [17]. Indeed, several failures of cardioprotection in human trials were foreshadowed by weak or negative findings in pigs, such as TRO40303 [1, 16] and cyclosporine [5, 20], aimed at mitochondrial membrane permeability, and cariporide, an inhibitor of the Na^+^/H^+^ exchanger [21, 29]. Likewise, a bolus of adenosine gave no protection in swine, paralleling the outcome in patients who were treated analogously [33]. Conversely, liraglutide, an analog of glucagon-like peptide-1, improved myocardial salvage in humans, despite negative results in pigs [4, 9].

Are there reasonable grounds to view the current study’s outcome as a possible false-negative? The considerations include drug levels, timing, drug action, and the injury model. Of these, pharmacokinetics appear unlikely. Inadequate stability in pigs and other reasons for inadequate drug levels were excluded, since the concentrations known to be effective in mouse myocardium and human cardiomyocytes [11] were achieved rapidly and exceeded in all animals. A U-shaped dose–response in vivo is theoretically possible, though not supported by the data in rodent and human cardiomyocytes [11]. MAP4K4 is identical across species including pig in its ATP-binding pocket, where DMX-5804, an ATP-competitive inhibitor, is known to bind; however, direct measurements of pig MAP4K4 ligand-binding and catalytic activity in the heart or non-cardiac tissues have not yet been undertaken. Because infarct size in the control group was no smaller than that produced by 60 min CAO in pentobarbital-anesthetized swine [7, 24], a confounding effect of sevoflurane (not started here until 15 min of reperfusion) is unlikely to have blunted the potential for cardioprotection. The study was adequately powered for precision of its end-points and expected effect size, making a type II error unlikely, and the magnitude of benefit was far less than postulated.

As one possible limitation, infarct size corrected for AAR was not only much greater than those provoked in mice [11], but also nearly twice those in some pig studies that also used 60 min of CAO [21, 23, 24, 28]; cf. [7, 22]. This difference is not conclusively explained, but likely relates to differences in the exact experimental conditions, including strain, anesthetic, open- versus closed-chest design, and even body temperature. It is unknown if factors underlying this disparity might make very large infarcts less remediable, at least by DMX-5804 and the MAP4K4 pathway. The median size of human infarcts after reperfusion is just 54–60% of AAR, as assessed by magnetic resonance imaging [1, 3]. Hence, it is worth posing the question if a large-mammal study is warranted whose control extent of injury is more similar to humans’, reducing the ischemic time or imposing a more distal occlusion. Supporting this interpretation, in hPSC-CMs, the extent of protection and half-maximal concentration for protection were dependent on the severity of oxidative stress [11]. Conceivably, cardioprotection might be enhanced by providing higher levels of DMX-5804 in the very first minutes after reperfusion, or by intracoronary delivery. However, the pro-drug was begun here 20 min before reperfusion, DMX-5804 was effective in mice even if administered systemically an hour afterwards, and the levels of DMX-5804 generated in pigs were at least tenfold higher than those in mice at the corresponding times after injury. Notably, the previous small-mammal data were no less rigorous than the present pig investigation: that study likewise was blinded and randomized [11], given known issues in the reproducibility of cardioprotective therapies [2, 19].

The advent of hPSC-CMs as a humanized preclinical platform is viewed with well-justified enthusiasm and was indeed a cornerstone of our drug development program. However, their track record for predicting eventual clinical success of novel therapies remains to be established, likely requiring many compounds’ systematic progression over the coming years. For now, it is a matter of conjecture whether studies in large mammals versus those in hPSC-CMs are more often “right” with respect to human biology, when findings in these platforms are discordant.

## Supplementary Information

Below is the link to the electronic supplementary material.

Supplementary file1 (DOCX 107 kb)

## Abbreviations

AAR: Area-at-risk
CAO: Coronary artery occlusion
CO: Cardiac output
${\text{d}}P/{\text{d}}t_{\max }$: Maximal rate of rise in LV pressure
${\text{d}}P/{\text{d}}t_{\min }$: Minimal rate of fall in LV pressure
hFABP: Heart-type fatty acid binding protein
hPSC-CMs: Human pluripotent stem cell-derived cardiomyocytes
HR: Heart rate
IA: Infarct area
IS: Infarct size
LV: LV
MAP: Mean arterial pressure
MAP4K4: Mitogen-activated protein kinase kinase kinase kinase-4
MI: Myocardial infarction
NR: No-reflow
OTW: Over-the-wire
SV: Stroke volume
SVR: Systemic vascular resistance
VF: Ventricular fibrillation
