# Supplementary material for: Preclinical trial of a MAP4K4 inhibitor to reduce infarct size in the pig: does cardioprotection in human stem cell-derived myocytes predict success in large mammals?
Source: Basic Res Cardiol. 2021 May 20;116(1):34. doi: 10.1007/s00395-021-00875-7 (PMC8137473; doi:10.1007/s00395-021-00875-7)
Supplement: Supplementary file 1 — Supplementary file1 (DOCX 107 kb) [file 395_2021_875_MOESM1_ESM.docx]

Preclinical trial of a MAP4K4 inhibitor to reduce infarct size in the pig: Does cardioprotection in human stem cell-derived myocytes predict success in large mammals?

Journal: Basic Research in Cardiology

Maaike te Lintel Hekkert, Gary Newton, Ph.D., Kathryn Chapman, Ph.D., Rehan Aqil, Ph. D., Robert Downham, Ph.D., Robert Yan, Ph.D., Daphne Merkus Ph.D., Gavin Whitlock, Ph.D., Charlotte A. L. Lane, Ph.D., Darren Cawkill, Ph.D., Trevor Perrior, Ph.D., Dirk J. Duncker, M.D., Ph.D.*, Michael D. Schneider, M.D.*

* Michael D. Schneider, Imperial College London, email [m.d.schneider@imperial.ac.uk](mailto:m.d.schneider@imperial.ac.uk); Dirk J. Duncker, Erasmus University Medical Center, email [d.duncker@erasmusmc.nl](mailto:d.duncker@erasmusmc.nl).

# supplemental materials

Online Tables 1-8.

Major Resources Table.

## Online Table 1: Effect of DMX-10001 on systemic hemodynamics and LV function, animal 1 (three-step infusion).

| **DMX-10001 (mg/kg/h):** | **41** | **15.3** | **9.8** |
| --- | --- | --- | --- |

|  | Baseline | 2 min | 10 min | 40 min | 1h | 2h | 3h | 4h | 6h | 8h | 8h 2 min | 8h 10 min | 8h 30 min |
| --- | --- | --- | --- | --- | --- | --- | --- | --- | --- | --- | --- | --- | --- |
| **Systemic hemodynamics** |  |  |  |  |  |  |  |  |  |  |  |  |  |
| HR (bpm) | 117 | 116 | 112 | 105 | 115 | 106 | 120 | 113 | 134 | 111 | 105 | 105 | 127 |
| MAP (mmHg) | 94 | 96 | 88 | 99 | 103 | 107 | 117 | 120 | 105 | 95 | 92 | 102 | 101 |
| CO (L/min) | 11.1 | 11.2 | 11.1 | 10.1 | 10.7 | 11.4 | 13.0 | 12.0 | 12.9 | 11.8 | 11.1 | 11.4 | 14.3 |
| SV (mL) | 95 | 97 | 99 | 96 | 93 | 108 | 108 | 106 | 96 | 106 | 106 | 109 | 113 |
| SVR (mmHg/L/min) | 8.5 | 8.6 | 7.9 | 9.8 | 9.7 | 9.4 | 9.0 | 10 | 8.2 | 8.0 | 8.3 | 8.9 | 7.1 |
| **Global LV function** |  |  |  |  |  |  |  |  |  |  |  |  |  |
| LV dP/dt_max_ | 3470 | 4070 | 3290 | 4060 | 4040 | 4840 | 6030 | 5260 | 5430 | 5020 | 4550 | 4730 | 5740 |
| LV dP/dt_min_ | -2500 | -2280 | -2280 | -2530 | -2690 | -3270 | -3330 | -3020 | -2830 | -2920 | -2820 | -3300 | -3190 |

A chronically instrumented animal received DMX-10001 for 8 h as shown, with a rapid initial infusion then stepwise decreasing concentrations over time. Hemodynamic and LV functional data were recorded throughout the infusion, until half an hour after its end. HR, heart rate; MAP, mean arterial pressure; CO, cardiac output; SV, stroke volume; SVR, systemic vascular resistance; dP/dt_max_, maximal rate of rise in LV pressure; dP/dt_min_, maximal rate of fall in LV pressure.

## Online Table 2: Effect of DMX-10001 on systemic hemodynamics and LV function, animal 2 (two-step infusion).

| **DMX-10001 (mg/kg/h):** | **60** | **15.3** |
| --- | --- | --- |

|  | **Baseline** | **2 min** | **10min** | **30 min** | **40 min** | **2h** | **24h** | **24h**  **2 min** | **24h**  **10 min** | **24h**  **30 min** | **25h** |
| --- | --- | --- | --- | --- | --- | --- | --- | --- | --- | --- | --- |
| **Systemic hemodynamics** |  |  |  |  |  |  |  |  |  |  |  |
| HR (bpm) | 101 | 116 | 90 | 92 | 83 | 91 | 107 | 111 | 87 | 92 | 115 |
| MAP (mmHg) | 77 | 80 | 83 | 84 | 90 | 94 | 89 | 72 | 97 | 144 | 153 |
| CO (L/min) | 7.8 | 9.7 | 6.9 | 6.7 | 6.1 | 7.2 | 8.7 | 9.1 | 7.0 | 7.7 | 9.1 |
| SV (mL) | 77 | 84 | 76 | 72 | 73 | 79 | 82 | 82 | 80 | 83 | 79 |
| SVR (mmHg/L/min) | 10.0 | 8.2 | 12.1 | 12.7 | 14.8 | 13.1 | 10.2 | 7.9 | 13.9 | 18.9 | 16.8 |
| **Global LV function** |  |  |  |  |  |  |  |  |  |  |  |
| LV dP/dt_max_ | 3800 | 5860 | 3400 | 3420 | 3680 | 4250 | 4260 | 5090 | 3950 | 4810 | 5360 |
| LV dP/dt_min_ | -3040 | -4170 | -3670 | -3430 | -3640 | -4120 | -3380 | -3740 | -4120 | -4140 | -4460 |

A chronically instrumented animal received DMX-10001 for 24 h as shown, starting with rapid infusion for half an hour then a lower concentration. Hemodynamic and LV functional data were recorded throughout the infusion, until half an hour after its end. HR, heart rate; MAP, mean arterial pressure; CO, cardiac output; SV, stroke volume; SVR, systemic vascular resistance; dP/dt_max_, maximal rate of rise in LV pressure; dP/dt_min_, maximal rate of fall in LV pressure.

**Online Table 3. Efficacy study enrollment summary and baseline data**

| \|  \| Prior to treatment \| Vehicle \| 1 \| \| --- \| --- \| --- \| --- \| | | | DMX-10001 |  | 1 |
| --- | --- | --- | --- | --- | --- | --- | --- | --- | --- |
| **Enrollment summary** | | |  |  |  |
| Animals entered | 5 | 15 | 16 |  |  |
| **Exclusions, by pre-defined exclusion criteria**  Convertible VF before onset occlusion | 1 | 0 | 0 |  |  |
| Non-convertible VF druring occlusion* | 2 | 0 | 0 |  |  |
| VF > 4 times before starting treatment | 0 | 0 | 0 |  |  |
| Developing pump failure during first 30 min of reperfusion | 0 | 0 | 0 |  |  |
| Incomplete coronary artery occlusion at end-CAO  Aberrant anatomy which prevent successful catheterization | 0 | 0 | 0 |  |  |
| AR (% of LV) < 15% | 0 | 0 | 1 |  |  |
| **Exclusions, other reasons**  Died during recovery day 1 | 0 | 0 | 1 |  |  |
| Found dead day 2 | 0 | 1 | 0 |  |  |
| Died during sedation day 2 | 0 | 1 | 1 |  |  |
| Technical failure  Malfunctioning coronary balloon catheter | 1 | 0 | 0 |  |  |
| Position coronary balloon changed during defibrillation | 1 | 0 | 0 |  |  |
| Staining failure^†^ | 0 | 1 | 1 |  |  |
| Statistical outlier | 0 | 0 | 1 |  |  |
| Final numer entered in analysis |  | 12 | 11 |  |  |
| **Baseline data** |  |  |  |  |  |
| Animal weight, kg | 40 ± 2 | | 42 ± 2 |  |  |
| Male/female | 6/6 | | 6/5 |  |  |

Values shown are n (enrollment summary) or the mean ± SEM (baseline data). * None of the animals developed non-convertible VF during reperfusion. ^†^ Staining difficulties compromising accurate measurement of AR.

## Online Table 4. Incidence of convertible VF and executed defibrillation shocks.

| **Convertible VF** | | **Vehicle** | **DMX-10001** |
| --- | --- | --- | --- |
| Occlusion 0-40 min | | 7 | 7 |
|  | No. of episodes | 1.1 ± 0.4 | 1.0 ± 0.0 |
|  | No. of defibrillation shocks | 3.4 ± 1.5 | 3.1 ± 1.6 |
| Occlusion 40-60 min | | 0 | 0 |
|  | No. of episodes | - | - |
| Reperfusion | | 0 | 0 |
|  | No. of episodes | - | - |

Values shown are n (incidence) or the mean ± SD (number of episodes or defibrillation shocks).

## Online Table 5. Hemodynamics, by fluid-filled catheter.

|  |  | |  | Coronary artery occlusion | | | Reperfusion  (day 1) | | | Reperfusion  (day 2) | |
| --- | --- | --- | --- | --- | --- | --- | --- | --- | --- | --- | --- |
|  |  | | Baseline | 15 min | 40 min | 60 min | 10 min | 30 min | 120 min | 22h 40 min | 23h 40 min |
|  |  | |  | **Start infusion 1** | | | **Start infusion 2** | | |  |  |
| HR (bpm) | | Vehicle | 129 ± 18 | 88 ± 25* | 83 ± 28* | 68 ± 20* | 87 ± 30* | 118 ± 36 | 107 ± 7 | 102 ± 11* | 98 ± 10* |
|  | | DMX-10001 | 124 ± 33 | 89 ± 40 | 78 ± 31* | 71 ± 21* | 99 ± 22 | 120 ± 31 | 94 ± 26 | 112 ± 24 | 103 ± 14 |
| MAP (mmHg) | | Vehicle | 81 ± 9 | 75 ± 23 | 67 ± 16 | 62 ± 13* | 76 ± 15 | 81 ± 13 | 73 ± 10 | 70 ± 12 | 72 ± 9 |
|  | | DMX-10001 | 89 ± 16 | 91 ± 30 | 71 ± 17 | 65 ± 11* | 74 ± 13 | 73 ± 11 | 73 ± 9 | 67 ± 13 | 75 ± 18 |
| SAP (mmHg) | | Vehicle | 101 ± 8 | 93 ± 21 | 86 ± 15 | 82 ± 13* | 92 ± 14 | 95 ± 13 | 88 ± 10* | 85 ± 14 | 87 ± 10 |
|  | | DMX-10001 | 107 ± 14 | 110 ± 34 | 88 ± 18 | 82 ± 13* | 91 ± 13 | 88 ± 11* | 87 ± 10* | 80 ± 13* | 91 ± 20 |
| DAP (mmHg) | | Vehicle | 67 ± 9 | 62 ± 21 | 54 ± 15 | 49 ± 12* | 63 ± 14 | 69 ± 12 | 62 ± 10 | 58 ± 10 | 60 ± 9 |
|  | | DMX-10001 | 75 ± 15 | 76 ± 28 | 58 ± 16 | 52 ± 10* | 61 ± 13 | 63 ± 11 | 62 ± 9 | 56 ± 13 | 62 ± 17 |

Data are shown as the mean ± SD. Vehicle, n = 12; DMX-10001, n = 11; * *P*<0.05 vs the corresponding treatment group baseline (2-way repeated measures ANOVA, followed by Bonferroni post-hoc testing).

## Online Table 6. LV dimensions and function, by echocardiography.

|  |  | |  | | | | |  | | | | | | |  |  |  |  |  |  |  |  |  |  |  |  |  |
| --- | --- | --- | --- | --- | --- | --- | --- | --- | --- | --- | --- | --- | --- | --- | --- | --- | --- | --- | --- | --- | --- | --- | --- | --- | --- | --- | --- |
|  | | | | **Day 1** | | | | | **Day 2** | | | |  |  |  |  |  |  |  |  |  |  |  |  |  |  |  |
|  | | | | **Baseline** | | | | | **End of Reperfusion** | | | |  |  |  |  |  |  |  |  |  |  |  |  |  |  |  |
| *Global LV function*  LV end-diastolic lumen surface (mm^2^) | | Vehicle | | | ­­  1559 | ± | 235 | | | 2005 | ± | 281 * | |  |  |  |  |  |  |  |  |  |  |  |  |  |  |
|  | | DMX-10001 | | | 1843 | ± | 287 | | | 1916 | ± | 460 | |  |  |  |  |  |  |  |  |  |  |  |  |  |  |
| LV end-systolic lumen surface (mm^2^) | | Vehicle | | | 858 | ± | 200 | | | 1433 | ± | 267 * | |  |  |  |  |  |  |  |  |  |  |  |  |  |  |
|  | | DMX-10001 | | | 1034 | ± | 174 | | | 1435 | ± | 381 * | |  |  |  |  |  |  |  |  |  |  |  |  |  |  |
| 2-D ejection fraction (%) | | Vehicle | | | 45 | ± | 9 | | | 28 | ± | 11 * | |  |  |  |  |  |  |  |  |  |  |  |  |  |  |
|  | | DMX-10001 | | | 44 | ± | 7 | | | 25 | ± | 9 * | |  |  |  |  |  |  |  |  |  |  |  |  |  |  |
| *Regional wall function (infarct region)*  LV end-diastolic wall thickness (mm) | | Vehicle | | | 8.2 | ± | 1.4 | | | 9.2 | ± | 1.5 * | |  |  |  |  |  |  |  |  |  |  |  |  |  |  |
|  | | DMX-10001 | | | 7.4 | ± | 1.2 | | | 8.5 | ± | 2.2 | |  |  |  |  |  |  |  |  |  |  |  |  |  |  |
| LV end-systolic wall thickness (mm) | | Vehicle | | | 11.6 | ± | 2.1 | | | 9.7 | ± | 1.6 * | |  |  |  |  |  |  |  |  |  |  |  |  |  |  |
|  | | DMX-10001 | | | 10.7 | ± | 1.9 | | | 8.7 | ± | 2.2 | |  |  |  |  |  |  |  |  |  |  |  |  |  |  |
| Systolic Wall thickening (infarct region, %) | | Vehicle | | | 44 | ± | 26 | | | 6 | ± | 9 * | |  |  |  |  |  |  |  |  |  |  |  |  |  |  |
|  | | DMX-10001 | | | 49 | ± | 30 | | | 3 | ± | 12 * | |  |  |  |  |  |  |  |  |  |  |  |  |  |  |
|  | | | | | | | | | | | | | | | | | | | |  |  |  |  |  |  |  |  |

Data are shown as the mean ± SD. Vehicle, n = 12; DMX-10001, n = 10 (1 of 11 was technically inadequate); * *P*<0.05 vs the corresponding baseline (2-way repeated measures ANOVA, followed by Bonferroni post-hoc testing).

## Online Table 7. LV function, by Millar catheter.

|  |  | | | | | |  | | | | | | | |  | | | | | | |  |  |  |  |  |
| --- | --- | --- | --- | --- | --- | --- | --- | --- | --- | --- | --- | --- | --- | --- | --- | --- | --- | --- | --- | --- | --- | --- | --- | --- | --- | --- |
|  | | | | | **Day 1** | | | | | | | | **Day 2** | | | | | | |  |  |  |  |  |  |  |
|  | | | | | **Baseline** | | | | | | | | **End of Reperfusion** | | | | | | |  |  |  |  |  |  |  |
| LV peak systolic pressure (mmHg) | | | Vehicle | | | 94 | | ± | | 10 | | 85 | | | | ± | | 13 | |  |  |  |  |  |  |  |
|  | | | DMX-10001 | | | 102 | | ± | | 16 | | 78 | | | | ± | | 15 * | |  |  |  |  |  |  |  |
| LV dP/dt_max_ (mmHg/s) | | | Vehicle | | | 1820 | | ± | | 250 | | 1090 | | | | ± | | 280 * | |  |  |  |  |  |  |  |
|  | | | DMX-10001 | | | 1830 | | ± | | 340 | | 920 | | | | ± | | 180 * | |  |  |  |  |  |  |  |
| LV dP/dt_min_ (mmHg/s) | | | Vehicle | | | -1830 | | ± | | 320 | | -1270 | | | | ± | | 330 * | |  |  |  |  |  |  |  |
|  | | | DMX-10001 | | | -2080 | | ± | | 460 | | -1240 | | | | ± | | 330 * | |  |  |  |  |  |  |  |
| LV dPdt_P40_ (mmHg/s) | | | Vehicle | | | 1750 | | ± | | 240 | | 1030 | | | | ± | | 270 * | |  |  |  |  |  |  |  |
|  | | | DMX-10001 | | | 1640 | | ± | | 320 | | 800 | | | | ± | | 220 * | |  |  |  |  |  |  |  |
| tau (ms) | | | Vehicle | | | 33 | | ± | | 4 | | 45 | | | | ± | | 10 * | |  |  |  |  |  |  |  |
|  | | | DMX-10001 | | | 34 | | ± | | 6 | | 44 | | | | ± | | 11 * | |  |  |  |  |  |  |  |
| LV end-diastolic pressure (mmHg) | | | Vehicle | | | 7 | | ± | | 3 | | 13 | | | | ± | | 5 * | |  |  |  |  |  |  |  |
|  | | | DMX-10001 | | | 8 | | ± | | 3 | | 13 | | | | ± | | 5 * | |  |  |  |  |  |  |  |
|  | |  | |  | | | | |  | |  | | |  | | |  | |  | |  |  |  |  |  |  |

LV dP/dt_max_, maximal rate of rise in LV pressure; LV dP/dt_min_, maximal rate of fall in LV pressure; tau = time constant of LV pressure decay. Data are shown as the mean ± SD. Vehicle, n = 12; DMX-10001, n = 11; * *P*<0.05 vs the corresponding baseline (2-way repeated measures ANOVA, followed by Bonferroni post-hoc testing).

## Online Table 8. Pressure-volume measurements, by conductance catheter.

|  |  |  | |  | |  | |  | |  | |  |  | | |  |
| --- | --- | --- | --- | --- | --- | --- | --- | --- | --- | --- | --- | --- | --- | --- | --- | --- |
|  | | | **End of Reperfusion** | | | | | | | | | | |  |  | |
|  | | | **Vehicle** | | | | | | **DMX-10001** | | | | |  | **P-value** | |
|  | | |  | |  | |  | |  | |  | |  |  |  | |
| Heart rate (beats/min) | | | 104 | | ± | | 14 | | 109 | | ± | | 18 |  | 0.531 | |
| Stroke volume (mL/beat) | | | 51 | | ± | | 16 | | 45 | | ± | | 15 |  | 0.341 | |
| Cardiac output (L/min) | | | 5.2 | | ± | | 1.4 | | 4.7 | | ± | | 1.3 |  | 0.378 | |
|  | | |  | |  | |  | |  | |  | |  |  |  | |
| LV end-diastolic pressure (mmHg) | | | 11 | | ± | | 5 | | 10 | | ± | | 3 |  | 0.785 | |
| LV peak-systolic pressure (mmHg) | | | 69 | | ± | | 8 | | 73 | | ± | | 17 |  | 0.499 | |
| tau (ms) | | | 27 | | ± | | 4 | | 28 | | ± | | 6 |  | 0.662 | |
|  | | |  | |  | |  | |  | |  | |  |  |  | |
| LV end-diastolic volume (mL) | | | 144 | | ± | | 43 | | 138 | | ± | | 52 |  | 0.754 | |
| LV end-systolic volume (mL) | | | 96 | | ± | | 33 | | 96 | | ± | | 47 |  | 0.996 | |
| 3-D ejection fraction (%) | | | 35 | | ± | | 8 | | 34 | | ± | | 13 |  | 0.734 | |
|  |  |  | |  | |  | |  | |  | |  |  | | |  |

Data are shown as the mean ± SD. Vehicle, n = 12; DMX-10001, n = 11; * *P*<0.05 (unpaired, two-tailed *t* test).

# Major Resources Table

## Animals (in vivo studies)

| **Species** | **Vendor or Source** | **Background Strain** | **Sex** | **Persistent ID / URL** |
| --- | --- | --- | --- | --- |
| Swine | Rovar Woensdrecht | Yorkshire x Landrace | M, F | https://www.roefsvarkens.nl |

## Other

| **Description** | **Source / Repository** | **Persistent ID / URL** |
| --- | --- | --- |
| Atropine sulphate | Teva Nederland BV, Haarlem, The Netherlands | https://www.teva.nl/ |
| Clinical guiding catheter (6F, JL3.5) | Medtronic, Minneapolis, MN | https://www.medtronic.com/ |
| Conductance catheter (7F) and software | CD Leycom, Hengelo, The Netherlands | http://www.cdleycom.eu/ |
| Data acquisition software (ATCODAS) | Dataq Instruments Inc., Akron OH | https://www.dataq.com/ |
| Dihydrostreptomycine sulphate | Instruvet Nederland bv, Boxmeer, The Netherlands | https://www.msd-animal-health.nl/ |
| Disposable silicone elastomeric infusion pump (Myfuser) | CANOX Medical Device S.R.L., Capurso, Italy | [info@canox.it](mailto:info@canox.it) (No URL available) |
| DMX-10001 | Domainex Ltd | https://www.domainex.co.uk/ |
| Echocardiograph (ZS3) | Mindray (formerly ZONARE Medical Systems Inc, Mountain View, CA) | https://www.mindraynorthamerica.com/ |
| EDTA-coated tubes | BD, Plymouth, UK | https://www.bd.com/ |
| Electric infusion pump (Alaris CC) | Cardinal Health, Role, Switzerland | https://www.cardinalhealth.com/ |
| Guide wire (ASAHI SION blue, 0.014”, 180 cm) | ASAHI Intecc co Ltd, Aichi, Japan | http://www.asahi-intecc.co.jp/en/ |
| Heparin | LEO Pharma, Amsterdam, The Netherlands | http://www.leo-pharma.nl/ |
| hFABP assay kits | Life Diagnostics, West Chester, PA | https://lifediagnostics.com/ |
| Iodixanol (Visipaque) | GE Healthcare BV, Eindhoven, The Netherlands |  |
| Isosorbidedinitrate (Cedocard) | Nycomed, Hoofddorp, The Netherlands | https://nycomedicalpharma.com/ |
| Li-heparin coated tubes | BD, Plymouth, UK | https://www.bd.com/ |
| MatLab | Mathworks Inc., Natick, MA | https://www.mathworks.com/ |
| Micro-manometer-tipped catheter (SPR-350S) | Millar Inc, Houston, TX | https://millar.com/ |
| Microplate photometer (Multiskan EX) | Thermo Scientific, Etten-Leur, The Netherlands | https://www.thermofisher.com/ |
| Midazolam | Allergan (formerly Actavis PLC, Baarn, The Netherlands) | https://www.allergan.com/ |
| Over-the-wire coronary angioplasty balloon (Sprinter OTW, 2-3 mm x 6 mm) | Medtronic, Minneapolis, MN | https://www.medtronic.com/ |
| Pharmacokinetic modeling | XenoGenesis, Alderley Park, UK | https://xenogesis.com/ |
| Pharmacokinetic profiling by liquid chromatography-mass spectrometry | Pharmidex, London, UK | https://www.pharmidex.com/ |
| Procainebenzyl penicillin | Instruvet Nederland bv, Boxmeer, The Netherlands | https://www.msd-animal-health.nl/ |
| Quantitative coronary angiography platform (CAAS) | PIE Medical, Maastricht, The Netherlands | https://www.piemedicalimaging.com/ |
| Sevoflurane | AbbVie BV, Hoofddorp, The Netherlands | https://www.abbvie.nl/ |
| Sufentanil (Sufenta forte) | Hameln Pharma plus GmbH, Hameln, Germany | https://www.hameln-group.com/ |
| Thioflavin-S | Sigma, Zwijndrecht, The Netherlands | https://www.sigmaaldrich.com/ |
| Thiopental sodium | PanPharma (formerly ROTEXMEDICA GmbH, Trittau, Germany) | https://www.panpharma.eu/de/ |
| Tiletamine/zolazepam (Zoletil50) | VIRBAC Nederland BV, Barneveld, The Netherlands | https://www.virbac.nl/ |
| Triphenyltetrazolium chloride | Sigma, Zwijndrecht, The Netherlands | https://www.sigmaaldrich.com/ |
| Xylazine (Sedazine10%) | AST Farma, Oudewater, The Netherlands | https://www.astfarma.nl/ |
